# Impact of infection on decongestion and kidney outcomes in patients with cardiorenal syndrome type 1

**DOI:** 10.1371/journal.pone.0355608

**Published:** 2026-08-07

**Authors:** Jonathan S. Chávez-Iñiguez, Jose J. Zaragoza, Ronaldo Escoto- Del Toro, Itzel Fong-Maravilla, Guillermo Navarro-Blackaller, Ramón Medina-González, Alejandro Martínez Gallardo-González, Luz Alcantar-Vallin, Juan A. Gómez-Fregoso, Eduardo Mendoza-Gaitán, Manuel Arizaga-Nápoles, Juan B. Ivey-Miranda, Guillermo García-García

**Affiliations:** 1 Nephrology Service, Hospital Civil de Guadalajara Fray Antonio Alcalde, Guadalajara, Jalisco, Mexico; 2 University of Guadalajara Health Sciences Center, Guadalajara, Jalisco, Mexico; 3 Intensive Care Unit, Hospital H + Queretaro, Santiago de Querétaro, Mexico; 4 Hospital de Cardiologia, Instituto Mexicano del Seguro Social, Mexico City, Mexico; Repubblica e Cantone Ticino Ente Ospedaliero Cantonale, SWITZERLAND

## Abstract

**Background:**

Infections are frequent precipitants of acute decompensated heart failure (ADHF) and may alter decongestive trajectories in patients with cardiorenal syndrome type 1 (CRS1), it may reduce diuretic efficacy and increase the risk of kidney injury. However, the impact of infection on decongestion and kidney outcomes in CRS1 remains unclear.

**Methods:**

We conducted a prospective cohort study including 256 patients with CRS1 hospitalized at a tertiary center (2022–2024). Patients were stratified by the presence of infection, defined as clinical suspicion plus antibiotic therapy. The primary outcome was successful decongestion, assessed by symptoms, biomarkers (BNP/CA-125), and POCUS findings. Secondary outcomes included major adverse kidney events at 10 and 30 days (MAKE: death, kidney replacement therapy [KRT], or ≥25% eGFR decline).

**Results:**

Seventy-two patients (28.1%) had infection, presenting with higher BNP (13,405 vs. 25,264 pg/mL, p = 0.012) and lower PaO_2_ (45 vs. 65.5 mmHg, p = 0.019). Furosemide exposure was comparable (600 vs. 580 mg, p = 0.62). Successful decongestion occurred in 61.4% of patients with infection vs. 59.8% without infection (p = 0.83). Infection was not independently associated with decongestion (aOR 1.28, 95% CI 0.65–2.51) or MAKE-30 (aOR 1.26, 95% CI 0.57–2.79).

**Conclusions:**

In CRS1, infection was associated with more severe baseline congestion but did not compromise decongestion rates or increase short-term MAKE. These findings support the notion that achieving decongestion is feasible in patients with ADHF due to infection without an incremental risk of MAKE.

## Introduction

Acute decompensated heart failure (ADHF) is a frequent cause of hospitalization and death among patients with cardiorenal syndromes (CRS) [1]. Common triggers include ischemic events, poor treatment adherence, lack of therapeutic optimization, arrhythmias, and infections among others [2–5]. Each of these precipitating factors may initiate distinct pathophysiological mechanisms, yet the majority of them ultimately converge in congestion and clinical deterioration [6]. Infections are a common cause of ADHF although the exact mechanisms remain unclear, involve multiple synergistic pathophysiological pathways, including endothelial dysfunction, systemic inflammation, neurohormonal activation, and hemodynamic alterations, ultimately resulting in sodium and water retention with subsequent congestion [2–5]. Despite the heterogeneous mechanisms underlying ADHF, the systematic approach to decongestion does not differentiate between etiological phenotypes of decompensation. It is possible that, due to its distinct pathophysiology, the trajectory and response to decongestion in patients with infection differ from those with other causes of ADHF, such as atrial fibrillation or treatment nonadherence.

Current international guidelines recommend decongestion of ADHF patients primarily through the use of diuretics [7], which should be titrated according to clinical response until effective decongestion is achieved [8]. However, nearly 25% of patients develop diuretic resistance, necessitating dose escalation or the addition of alternative strategies to optimize decongestion [9]. Diuretics may be less effective in patients with active infection, since systemic inflammation, endothelial dysfunction, and sepsis-related hemodynamic alterations can impair renal perfusion and reduce natriuretic response. Furthermore, infection-induced activation of neurohormonal pathways and cytokine release may promote sodium and water retention, thereby increasing the risk of diuretic resistance and attenuating the efficacy of standard decongestive strategies [10,11]. Identifying differential responses to treatment in CRS patients with congestion according to the presence of infection would be of clinical value, as it may allow the early recognition of distinct phenotypes and the anticipation of tailored therapeutic strategies. To address this knowledge gap, we aimed to investigate the rate of successful decongestion CRS patients according to the presence of infection, as well as their risk of developing major adverse kidney events (MAKE) during follow-up.

## Methods

### Study design and patient population

The present study was an investigator-initiated prospective cohort conducted at the Hospital Civil de Guadalajara Fray Antonio Alcalde, Guadalajara, Mexico. Potential participants were identified during routine hospital rounds of patients with acute kidney injury (AKI), and eligibility was confirmed through concurrent review of clinical records. Patients diagnosed with cardiorenal syndrome type 1 (CRS1) were included in the study, CRS1 was defined according to the 2008 classification system by Ronco et al, and both AKI and ADHF criteria needed to be present at baseline [12]. Cardiology and nephrology teams confirmed the presence of CRS1. ADHF was clinically defined [13], AKI was made using the serum creatinine (sCr) by KDIGO criteria [14]. The eGFR was calculated according to the Chronic Kidney Disease Epidemiology Collaboration (CKD-EPI) equation [15].

Infection was defined as the presence of a clinical suspicion of bacterial infection (pulmonary, abdominal, urinary, soft tissue, or other sites) combined with the prescription of antibiotics. MAKE outcomes were defined as death, a new requirement for KRT, or worsening kidney function by a ≥ 25% decline in the eGFR from baseline, [16] and they were evaluated during the first 10 days (MAKE10) and at 30 days (MAKE 30). We followed the 31st Acute Disease Quality Initiative group recommendations on the design of studies to explore treatments for patients with AKI and selected the sub-phenotype of patients with CRS and infection [17]. In addition, to better capture the interaction between kidney trajectory and decongestion, the cohort was stratified based on two key clinical parameters: presence or absence of infection as the main cause of decompensation of ADHF, and achievement or failure of clinical decongestion, evaluated through a composite assessment including symptom resolution, improvement in biomarkers (such as BNP or CA-125), and POCUS findings (e.g., lung ultrasound, IVC status, and VExUS score). Successful decongestion was defined as at least 1 of the following metrics: resolution of dyspnea and peripheral edema, > 30% reduction in BNP levels, and absence of B-lines or VExUS ≥ 2. The diuretics management was at the discretion of both teams according to institutional standards.

Inclusions criteria were: (1) clinical diagnosis of ADHF; (2) AKI as per KDIGO criteria on admission [14]; (3) availability of baseline sCr in the 6 months prior to hospitalization; and (4) at least one follow-up sCr measurement within 30 days. Patients were excluded if they had had AKI within the past three months, were <18 years old, had CKD grade 5, chronic dialysis, kidney transplant, hospital stay <48 hours, or had missing data that would render analysis incomplete. The main exposure was the presence of infection and its association with effective decongestion and MAKE.

### Data collection

Clinical characteristics, demographic information, and laboratory data were collected via automated retrieval from the institutional electronic medical records system (10 October 2025). We also considered other potential contributing factors to AKI, including nephrotoxic drugs such as aminoglycosides, non-steroidal anti-inflammatory drugs, and vancomycin. The indications for KRT included persistent congestion that was resistant to diuretics, severe hyperkalemia, severe metabolic acidosis, and uremic manifestations, such as encephalopathy, pericarditis, and seizures [18]*.* This was an exploratory analysis; hence, no formal sample size calculation was performed. However, the number of events was deemed sufficient for the planned multivariable analyses (≥10 events per variable).

This study was approved by the Hospital Civil de Guadalajara Fray Antonio Alcalde Institutional Review Board (IRB HCG/CEI-0550/15) and was conducted according to the Declaration of Helsinki. Patient consent was not required in accordance with national guidelines, and the IRB approved a waiver of informed consent in accordance with local regulations. The study protocol was designed to align with the Strengthening the Reporting of Observational Studies in Epidemiology (STROBE) guidelines [19] and the REporting of studies Conducted using Observational Routinely collected health Data (RECORD) statement [20].

### Study objectives

The primary outcome was successful decongestion in CRS1 patients stratified by the presence of infection. Secondary outcomes were MAKE-30 and their separate subcomponents as Mortality, KRT and Worsening Kidney Function also stratified by the presence of infection.

### Statistical analysis

Baseline demographic, clinical, and laboratory characteristics were summarized for the total cohort and stratified by the presence or absence of infection. Continuous variables were assessed for normality and presented as median and interquartile range (IQR) as all variables followed a non-normal distribution. Comparisons between groups were made using the Mann-Whitney U test. Categorical variables were presented as frequencies and percentages (%), and comparisons were performed using the Chi-squared test or Fisher’s exact test, as appropriate. To assess the independent association between infection (exposure) and the study outcomes, we performed multivariable logistic regression analysis. The primary outcome was successful decongestion, and the main secondary outcome was the incidence of MAKE-30. Infection was analyzed as an independent variable and was coded as a binary. For each outcome, we developed a primary multivariable model adjusted for a set of clinically relevant confounders selected a priori: age, sex, history of congestive heart failure, history of CKD, and baseline creatinine. To assess the robustness of our findings, we conducted a sensitivity analysis by developing a second, more comprehensive model. This sensitivity model included the variables from the primary model plus additional baseline covariates that were significantly different between groups in the descriptive analysis (p < 0.1), namely respiratory rate, leukocyte count, and glucose. The results of the logistic regression models are presented as adjusted Odds Ratios (aOR) with their corresponding 95% confidence intervals (95% CI). Complete-case analysis was performed for all multivariable models. Patients with missing values in any covariate included in a given model were excluded from that specific analysis. The reduction in sample size of the decongestion model was mainly attributable to missing biomarker and imaging variables that were obtained according to clinical availability rather than systematically in all participants. For the MAKE-30 analyses, only patients with complete 30-day follow-up and complete outcome ascertainment were included to avoid misclassification of the composite endpoint. Because missingness primarily reflected incomplete follow-up rather than isolated missing baseline covariates, multiple imputation was not considered methodologically appropriate. Finally, the time to MAKE was visualized using Kaplan-Meier curves, and the difference between the infection and no-infection groups was formally compared using the log-rank test. Patients with missing time-to-event data were excluded from this specific analysis.

All statistical analyses were performed using Stata version 16.0 (StataCorp, College Station, TX, USA). A two-sided p-value < 0.05 was considered statistically significant for all analyses.

## Results

**Fig 1:**
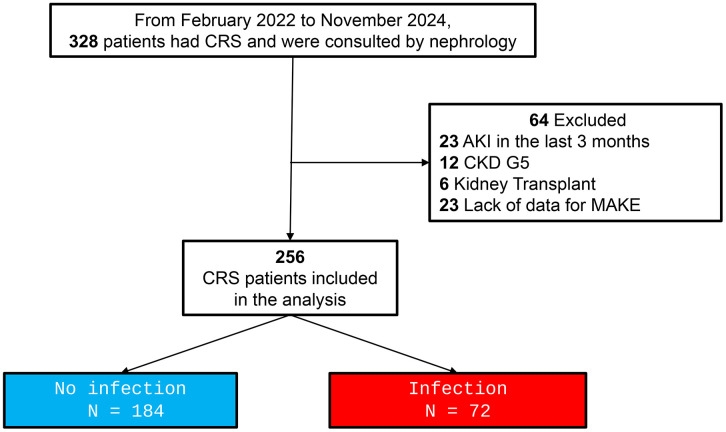
Flowchart of study population.

From February 2022 to November 2024, a total of 328 patients with CRS were assessed by the nephrology service. Sixty-four subjects were excluded (Fig 1), and the final cohort included 256 CRS patients. Of these 72 (28.1%) were diagnosed with a concurrent infection, while 184 (71.9%) constituted the non-infected control group as shown in the flow chart of Fig 1.

Demographic and clinical characteristics of CRS patients according to the infection status group are presented in Table 1. Median age was similar between groups (65 vs. 64.5 years, *p* = 0.51), and males represented 55.6% of the infection group (*p* = 0.99). Likewise, the prevalence of diabetes, systemic hypertension, CKD, and congestive heart failure did not differ significantly, indicating that both groups were similar at baseline. As expected, infected patients showed higher antibiotic exposure (100% vs. 39.9%, *p* < 0.001), and non-steroidal anti-inflammatory drugs (NSAID) use was more frequent (19.4% vs. 9.8%, *p* = 0.037). Conversely, SGLT2 inhibitor use was significantly less common among those with infection (41.3% vs. 64.3%, *p* = 0.002), which may reflect treatment discontinuation during acute illness or differential prescribing practices. Markers of anemia and inflammation were also more pronounced in the infection group, with lower hemoglobin (9.77 vs. 11.1 g/dL, *p* = 0.022) and hematocrit (30.1% vs. 34.2%, *p* = 0.011). Importantly, markers of congestion were significantly worse in patients with infection, as they had markedly higher BNP concentrations (Q1 25,264 vs. Q3 13,405 pg/mL, *p* = 0.012) and significantly lower arterial oxygen tension (pO_2_ 45 vs. 65.5 mmHg, *p* = 0.019), likely indicating more severe pulmonary congestion and impaired gas exchange. The cumulative furosemide dose was similar between groups, with a median of 580 mg (IQR 330–920) in patients without infection and 600 mg (IQR 350–940) in those with infection, with no statistically significant difference. The proportion of patients requiring KRT specifically for volume overload was comparable (20.8% vs. 17.4%, *p* = 0.52).

**Table 1:** Baseline demographic and clinical characteristics of patients according to the presence of infection.

Variable	No infection n = 184	Infection n = 72	Overall cohort n = 256	p
Age	64.5 (53.5-74)	65 (52-73.5)	65 (53-74)	0.51
Male sex	102 (55.4%)	40 (55.6%)	142 (55.5%)	0.99
Diabetes Mellitus	113 (61.4%)	46 (63.9%)	159 (62.1%)	0.71
Hypertension	128 (69.6%)	47 (65.3%)	175 (68.4%)	0.51
Tobacco use	34 (18.5%)	16 (22.2%)	50 (19.5%)	0.5
Hypothyroidism	14 (7.6%)	4 (5.6%)	18 (7.0%)	0.56
CHF	114 (62.0%)	46 (63.9%)	160 (62.5%)	0.77
CKD	99 (54.1%)	33 (45.8%)	132 (51.8%)	0.23
Previous stroke	6 (3.3%)	4 (5.6%)	10 (3.9%)	0.39
Ischemic heart disease	51 (27.7%)	13 (18.1%)	64 (25.0%)	0.11
Cirrhosis	2 (1.1%)	1 (1.4%)	3 (1.2%)	0.84
NSAIDs	18 (9.8%)	14 (19.4%)	32 (12.5%)	0.037*
Antibiotic use	73 (39.9%)	72 (100%)	145 (56.6%)	<0.001*
Antihypertensive	131 (71.6%)	47 (65.3%)	178 (69.8%)	0.32
Diuretic	166 (90.7%)	56 (77.8%)	222 (87.1%)	0.006*
Vasopressor	30 (16.4%)	16 (22.2%)	46 (18.0%)	0.28
Statin use	112 (61.2%)	37 (51.4%)	149 (58.4%)	0.15
Acetylsalicylic acid	67 (36.6%)	15 (20.8%)	82 (32.2%)	0.015*
SGLT2i	110 (64.3%)	26 (41.3%)	136 (58.1%)	0.002*
Weight, kg	69 (60-82)	70 (59.8-81)	70 (60-81)	0.68
Systolic blood pressure	120 (106-136)	116.5 (100-131)	119 (105-135)	0.32
Diastolic blood pressure	70 (60-80)	70 (60-77.5)	70 (60-80)	0.37
Heart rate	80 (72-94)	81 (70-90)	80 (72-92.5)	1
Respiratory rate	19.5 (16-20)	20 (18-21.5)	20 (16-20)	0.038*
Temperature	36.5 (36-36.5)	36.40 (36-36.6)	36.5 (36-36.5)	0.64
KRT received				
Hemodialysis	23 (12.5%)	12 (16.7%)	35 (14.7%)	0.48
Peritoneal Dialysis	7 (3.8%)	6 (8.4%)	13 (5.1%)	
Medical treatment	140 (76.1%)	52 (72.2%)	192 (75.0%)	
CRRT	14 (7.6%)	2 (2.8%)	16 (6.3%)	
Total Furosemide dose (mg)	580 (330-920)	600 (350-940)	590 (330-940)	0.62
Indication for KRT				
Hyperkalemia	12 (6.5%)	7 (9.7%)	19 (7.4%)	0.38
Metabolic acidosis	10 (5.4%)	3 (4.2%)	13 (5.1%)	0.68
Volume overload	32 (17.4%)	15 (20.8%)	47 (18.4%)	0.52
Uremia	15 (8.2%)	7 (9.7%)	22 (8.6%)	0.69
AKI Stage 1	14 (7.6%)	3 (4.2%)	17 (6.6%)	0.47
AKI Stage 2	23 (12.5%)	7 (9.7%)	30 (11.7%)	
AKI Stage 3	147 (79.9%)	62 (86.1%)	209 (81.6%)	
Baseline sCr	1.5 (.89-2.2)	1.5 (.75-2.7)	1.5 (.86-2.36)	0.88
Baseline eGFR	67.5 (30-100)	82 (25-100)	77 (30-100)	0.65
Hypochloremia	54 (30.9%)	18 (25.7%)	72 (29.4%)	0.42
Hemoglobin (g/dl)	11.1 (9.32-13.13)	9.77 (8.31-13.08)	10.825 (9.03-13.08)	0.022*
Hematocrit (%)	34.2(29.12-39.40)	30.09 (25.82-39.6)	33.4 (28.28-39.40)	0.011*
Platelets, U/L	214 (156-282)	193 (129-263)	207 (148-281)	0.15
Leucocytes	9.58 (7.46-13.36)	11.17 (8.32-15.25)	10.215 (7.51-13.77)	0.094
Glucose (mg/dl)	95.5 (76.5-139)	113 (84-157)	100 (77-145)	0.061
Urea (mg/dl)	138.5 (100-184)	156 (113-200.60)	144 (102.7-185)	0.19
Creatinine (mg/dl)	3.11 (2.39-4.65)	3.46 (2.65-5.15)	3.21 (2.42-4.92)	0.16
Albumin (g/l)	3.04 (2.40-3.47)	2.83 (2.49-3.21)	3 (2.42-3.40)	0.36
Total proteins (g/l)	5.90 (5.17-6.60)	5.90 (5.40-6.30)	5.90 (5.30-6.60)	0.8
Sodium (mEq/L)	135 (132-138)	135 (132-138)	135 (132-138)	0.88
Potassium (mEq/L)	4.78 (4.10-5.46)	4.5 (4-5.42)	4.70 (4.06-5.46)	0.18
Chloride (mEq/L)	102 (96-106)	102 (97-106)	102 (97-106)	0.85
Calcium (mg/dl)	8.20 (7.70-8.80)	8 (7.55-8.5)	8.20 (7.60-8.60)	0.072
Phosphorus (mg/dl)	4.90 (4.10-6.20)	5 (4.01-5.90)	5 (4.01-6.01)	0.9
B-natriuretic peptide (pg/mL)	13405 (8354-22864)	25264 (14044-33787)	16862 (8782-24988)	0.012*
CA-125	375.2 (264.5-423.1)	676.7 (87.4-1266)	375.2 (164.9-459.9)	1
Proteinuria	30 (0-100)	100 (30-100)	30 (0-100)	0.22
Hematuria	80 (5-200)	200 (80-200)	80 (25-200)	0.15
pH	7.34 (7.29-7.41)	7.36 (7.34-7.42)	7.35 (7.31-7.41)	0.31
pCO2	37 (29-46)	38 (31-45)	37 (29-46)	0.89
pO2	65.5 (47.5-92)	45 (40-73)	59 (43-91)	0.019*
Bicarbonate (mmol/L)	20.30 (15.6-26.6)	19.5 (17.1-23.4)	20.15 (16.1-26.6)	0.94
Lactate	1.55 (.90-2.35)	1.5 (1.1-2.10)	1.5 (.90-2.3)	0.88

Abbreviations: AKI: acute kidney injury, BMI: Body mass index; CHF: chronic heart failure, CKD: chronic kidney disease, eGFR: Estimated Glomerular filtration rate; Hb: Hemoglobin; KRT: Kidney replacement therapy; NSAIDs: non-steroidal analgesic autoinflammatory drugs, sCr: serum creatinine; SGLT2i sodium glucose type 2 inhibitors.

### Primary outcome: Successful decongestion in CRS patients according to the presence of infection

**Fig 2:**
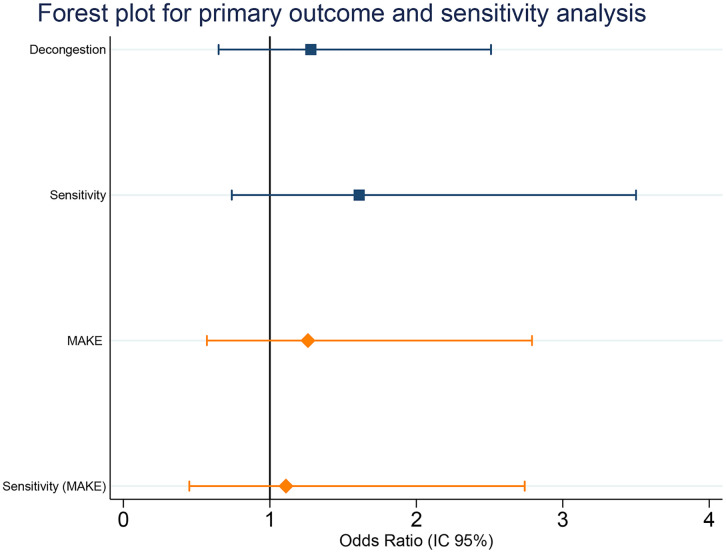
Forest plot of adjusted odds ratios for the primary and secondary outcomes.

Successful decongestion was achieved in 107 patients without infection (59.8%) and in 43 patients with infection (61.4%), with no significant difference between groups (p = 0.83). The frequency of decongestion according to the site of infection is described in the S1 Fig. The multivariable decongestion model included 198 patients with complete covariate information, whereas the adjusted MAKE-30 model included 134 patients with complete 30-day follow-up and complete outcome ascertainment. The primary analysis demonstrated that infection was not independently associated with lower odds of achieving decongestion (adjusted OR 1.28, 95% CI 0.65–2.51, *p* = 0.48) Table 2. Similarly, in the sensitivity model incorporating additional clinical and biochemical parameters such baseline respiratory rate, leukocyte count, and glucose, the association remained non-significant (adjusted OR 1.61, 95% CI 0.74–3.50, *p* = 0.23). A visual summary of the adjusted OR for successful decongestion is presented in the forest plot in Fig 2.

**Table 2:** Multivariate model and sensitivity analysis for primary and secondary outcomes.

	Adjusted Odds Ratio (95% CI)	p-value
Primary Outcome: Decongestion		
Primary Model (N = 198)		
Infection (ref: No)	1.28 (0.65 - 2.51)	0.48
Age	1.02 (0.99 - 1.04)	0.13
Sex (ref: Female)	0.92 (0.50 - 1.68)	0.79
Congestive Heart Failure	1.60 (0.86 - 2.99)	0.14
Chronic Kidney Disease	1.01 (0.46 - 2.21)	0.98
Baseline Creatinine	0.88 (0.61 - 1.27)	0.50
Sensitivity Model (N = 169)		0.00
Infection (ref: No)	1.61 (0.74 - 3.50)	0.23
Age	1.01 (0.99 - 1.04)	0.19
Sex (ref: Female)	1.00 (0.50 - 1.99)	0.99
Congestive Heart Failure	1.55 (0.77 - 3.14)	0.22
Chronic Kidney Disease	0.95 (0.40 - 2.27)	0.91
Baseline Creatinine	0.93 (0.61 - 1.40)	0.72
Respiratory Rate	1.02 (0.93 - 1.12)	0.69
Leukocyte count	0.99 (0.93 - 1.05)	0.73
Glucose	1.00 (0.99 - 1.00)	0.14
Secondary Outcome: MAKE-30		
Primary Model (N = 134)		
Infection (ref: No)	1.26 (0.57 - 2.79)	0.57
Age	1.00 (0.98 - 1.02)	0.85
Sex (ref: Female)	1.09 (0.54 - 2.21)	0.81
Congestive Heart Failure	1.13 (0.55 - 2.33)	0.73
Chronic Kidney Disease	1.03 (0.42 - 2.54)	0.95
Baseline Creatinine	1.12 (0.74 - 1.69)	0.60
Sensitivity Model (N = 111)		0.00
Infection (ref: No)	1.11 (0.45 - 2.74)	0.82
Age	1.00 (0.98 - 1.03)	0.80
Sex (ref: Female)	0.89 (0.41 - 1.97)	0.78
Congestive Heart Failure	1.08 (0.48 - 2.44)	0.85
Chronic Kidney Disease	1.09 (0.40 - 3.01)	0.87
Baseline Creatinine	1.29 (0.81 - 2.07)	0.29
Respiratory Rate	1.06 (0.95 - 1.19)	0.30
Leukocyte count	1.05 (0.98 - 1.13)	0.15
Glucose	1.00 (0.99 - 1.00)	0.79

aOR: adjusted Odds Ratio; 95% CI: 95% Confidence Interval; MAKE-30: Major Adverse Kidney Events at 30 days.

The Primary Model was adjusted for age, sex, congestive heart failure, chronic kidney disease, and baseline creatinine.

The Sensitivity Model was adjusted for all variables in the primary model plus baseline respiratory rate, leukocyte count, and glucose.

### Secondary outcomes

**Fig 3:**
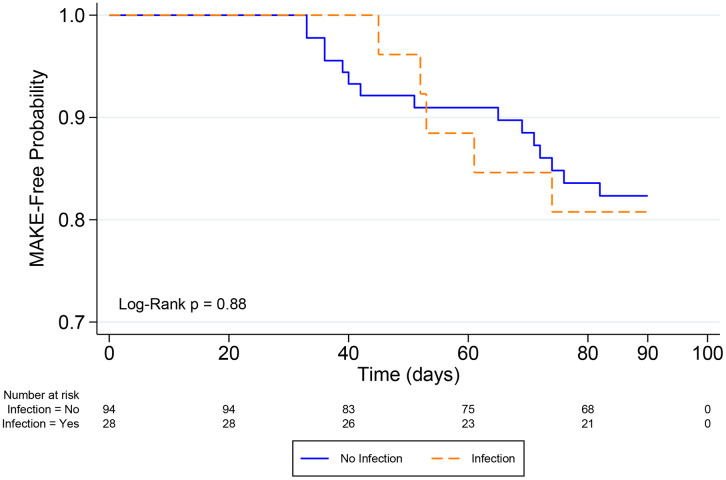
Kaplan–Meier analysis of MAKE-free survival according to infection status. Kaplan–Meier curves demonstrating the probability of remaining free from MAKE during follow-up in patients with and without infection. The curves remained largely overlapping throughout follow-up, indicating no significant difference in the cumulative incidence of MAKE between groups (log-rank p = 0.88). Numbers at risk are shown below the x-axis.

**Fig 4:**
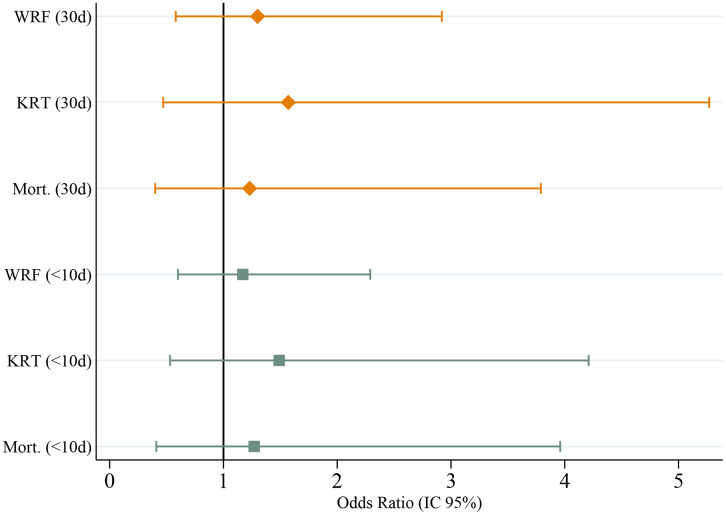
Forest plot of adjusted odds ratios for subcomponents of MAKE.

The secondary objectives are presented in Table 2 and 3. MAKE-10 occurred in 151 patients without infection (82.1%) and in 65 patients with infection (90.3%), with no statistically significant difference between groups (p = 0.13). MAKE-30 occurred in 59 patients without infection (43.1%) and in 23 patients with infection (50.0%), with no significant difference between groups (p = 0.45). In the primary model, infection did not increase the risk of MAKE-30 (adjusted OR 1.26, 95% CI 0.57–2.79, *p* = 0.57), and this finding was consistent in the sensitivity model (adjusted OR 1.11, 95% CI 0.45–2.74, *p* = 0.82). Similarly, demographic characteristics such as age and sex were not associated with MAKE-30, nor were comorbid conditions including CHF and CKD. Additional relevant clinical and laboratory parameters, including respiratory rate, leukocyte count, and glucose, also failed to demonstrate independent predictive value, Table 2. Fig 3 shows the Kaplan–Meier curves for MAKE-free survival according to infection status, demonstrating no significant difference between groups throughout follow-up (log-rank p = 0.88). The analysis of MAKE subcomponents revealed that infection was not independently associated with short-term mortality, initiation of KRT, or worsening kidney function in CRS patients. At 10 days, adjusted odds ratios for infection showed no significant association with mortality (aOR 1.27, 95% CI 0.41–3.96, *p* = 0.687), KRT initiation (aOR 1.49, 95% CI 0.53–4.21, *p* = 0.453), or worsening kidney function (aOR 1.17, 95% CI 0.60–2.29, *p* = 0.647). These findings were consistent at 30 days, where infection again failed to reach significance across mortality (aOR 1.23, 95% CI 0.40–3.79, *p* = 0.713), KRT initiation (aOR 1.57, 95% CI 0.47–5.27, *p* = 0.463), and worsening kidney function (aOR 1.30, 95% CI 0.58–2.92, *p* = 0.524); See Table 3 and Fig 4. Other covariates, including age, sex, congestive heart failure, CKD, and baseline creatinine, did not demonstrate significant associations with any MAKE subcomponent, although trends were observed.

**Table 3:** Multivaritae analysis for MAKE componentes.

	Mortality (<10d)	KRT Initiation (<10d)	Worsening Kidney Function
	aOR (95% CI)	aOR (95% CI)	aOR (95% CI)
Infection (ref: No)	1.27 (0.41 - 3.96)	1.49 (0.53 - 4.21)	1.17 (0.60 - 2.29)
	p = 0.687	p = 0.453	p = 0.647
Age	1.01 (0.98 - 1.04)	0.99 (0.96 - 1.02)	1.00 (0.98 - 1.02)
	p = 0.462	p = 0.485	p = 0.910
Sex (ref: Female)	0.93 (0.32 - 2.68)	1.25 (0.50 - 3.14)	0.81 (0.43 - 1.54)
	p = 0.887	p = 0.630	p = 0.518
Congestive Heart Failure	1.34 (0.46 - 3.88)	1.24 (0.48 - 3.20)	1.01 (0.53 - 1.93)
	p = 0.592	p = 0.655	p = 0.967
Chronic Kidney Disease	1.15 (0.33 - 4.02)	1.48 (0.50 - 4.39)	1.05 (0.54 - 2.05)
	p = 0.824	p = 0.480	p = 0.880
Baseline Creatinine	1.04 (0.75 - 1.45)	1.15 (0.83 - 1.60)	1.05 (0.84 - 1.32)
	p = 0.796	p = 0.410	p = 0.669
			
	**Mortality (30d)**	**KRT Initiation (30d)**	**Worsening Kidney Function**
	aOR (95% CI)	aOR (95% CI)	aOR (95% CI)
Infection (ref: No)	1.23 (0.40 - 3.79)	1.57 (0.47 - 5.27)	1.30 (0.58 - 2.92)
	p = 0.713	p = 0.463	p = 0.524
Age	1.01 (0.98 - 1.05)	0.99 (0.96 - 1.03)	1.00 (0.98 - 1.02)
	p = 0.518	p = 0.638	p = 0.988
Sex (ref: Female)	1.10 (0.38 - 3.19)	1.31 (0.43 - 4.01)	0.97 (0.48 - 1.95)
	p = 0.858	p = 0.637	p = 0.923
Congestive Heart Failure	1.40 (0.49 - 3.99)	1.15 (0.36 - 3.65)	1.14 (0.56 - 2.31)
	p = 0.526	p = 0.806	p = 0.730
Chronic Kidney Disease	1.04 (0.28 - 3.84)	1.63 (0.48 - 5.57)	1.02 (0.49 - 2.11)
	p = 0.954	p = 0.435	p = 0.966
Baseline Creatinine	1.07 (0.76 - 1.49)	1.18 (0.83 - 1.68)	1.05 (0.83 - 1.34)
	p = 0.697	p = 0.360	p = 0.677

All models are adjusted for age, sex, congestive heart failure, chronic kidney disease, and baseline creatinine. aOR: adjusted Odds Ratio; 95% CI: 95% Confidence Interval; RRT: Renal Replacement Therapy.

In an exploratory analysis, we investigated a potential interaction between infection status and vasopressor use on study outcomes. The study population was stratified into four groups: no infection/no vasopressor (n = 153), no infection/vasopressor (n = 30), infection/no vasopressor (n = 56), and infection/vasopressor (n = 16). A significant difference was found in the proportion of patients receiving diuretics across these groups (p = 0.030, Fisher’s exact test), with the lowest proportion observed in the infection/no vasopressor group (76.8%). In the adjusted logistic regression model for successful decongestion, a statistically significant interaction between infection and vasopressor use was detected (p for interaction = 0.020). The aOR for the infection/ vasopressor group was 0.13 (95% CI 0.02–0.72), suggesting that infection significantly reduced the odds of successful decongestion in patients receiving vasopressors. Conversely, in the adjusted model for MAKE-30, the interaction term was not statistically significant (OR 7.67, 95% CI 0.46–127.1 p = 0.155), likely due to the small sample size in the infection/vasopressor group. These exploratory findings should be interpreted with caution due to the limited statistical power for interaction analyses in this cohort.

## Discussion

**Fig 5:**
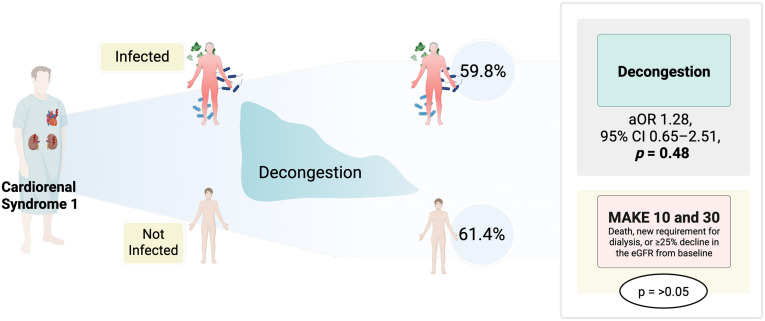
Central image.

These findings indicate that, although CRS1 infected patients presented with higher markers of vascular congestion at baseline, its presence per se did not preclude the likelihood of achieving decongestion during follow-up. (Fig 5: Central Image)

While prior studies have consistently demonstrated that infections precipitate a large proportion of ADHF cases and worsen short-term prognosis, mainly by increasing in-hospital mortality [2. 3, 4], none specifically evaluated the impact of infection on the process of decongestion or the development of MAKE. In contrast, in our cohort of patients with CRS1, those presenting with infection exhibited more severe congestion at baseline, as reflected by higher BNP levels and impaired oxygenation. However, after multivariable adjustment, infection was not independently associated with reduced odds of achieving successful decongestion or with MAKE. These findings suggest that, although infection clearly contributes to worse cardiovascular outcomes in broader ADHF populations, in CRS1 patients its influence on short-term renal and decongestion outcomes may be less direct, being modulated instead by comorbidities, baseline kidney function, and hemodynamic status. Thus, our study provides complementary evidence that infection-related decompensations do not necessarily preclude effective decongestion or predict early adverse kidney events in this specific population.

Previous studies have consistently identified infections as a common precipitant of ADHF, accounting for approximately 25% of hospitalizations and being associated with higher in-hospital mortality, prolonged length of stay, and worse functional outcomes [21–24]. As in our study, respiratory infections are the most frequent subtypes [22]. Our results therefore complement prior evidence by underscoring the need to refine CRS1 phenotyping and to evaluate whether infection-related decompensations represent a distinct subgroup with differential trajectories and treatment responses.

Current evidence indicates that in patients with ADHF and active infection, diuretics remain the cornerstone of decongestive therapy, with dosing tailored according to early natriuretic response, kidney function trajectory, and hemodynamic status [25–30]. Intensification with higher doses or the addition of thiazides or acetazolamide is recommended in the setting of suboptimal response, while close monitoring is essential given the increased risk of hypotension, AKI, and electrolyte disturbances, particularly in sepsis or shock [26–28]. Ultrafiltration is generally reserved for refractory cases [30,31]. The concern regarding whether diuretics are equally safe and effective in patients with CRS1 and concomitant infection is justified, as diuretics have been reported to exert nephrotoxic effects [32] and to increase the incidence of AKI when administered during hospitalization [33,34]. Specifically, in the CRS setting, decongestion has been associated with “permissive” increases in serum creatinine that may not necessarily reflect true kidney injury but rather hemodynamic adaptation [35]. Moreover, their hemodynamic impact on mean arterial pressure in critically ill patients appears minimal [36]. In our exploratory sub-analysis the observed interaction between infection and vasopressor use may reflect an amplified state of hemodynamic vulnerability in which competing physiologic priorities limit effective decongestion. In the presence of infection and shock, vasopressor therapy redirects perfusion toward vital organs at the expense of renal blood flow. This combination may narrow the “hemodynamic window” for diuretic responsiveness, thereby reducing the likelihood of achieving successful decongestion. Importantly, this interaction did not extend to MAKE-30, likely due to limited power in this subgroup rather than absence of biological plausibility.

Additionally, restricted to patients with infection, we observed variability in the rates of successful decongestion depending on the infection source, with the lowest rate seen among those with urinary tract infections and the highest in the miscellaneous ‘Others’ group. Although these differences were not statistically significant, the pattern raises interesting considerations. Prior studies in ADHF have consistently reported that respiratory infections and sepsis/bacteremia are the most frequent precipitants and are associated with worse short-term outcomes, including mortality and rehospitalization [22–24]. However, evidence regarding the differential impact of infection sites on decongestion or kidney-specific outcomes remains scarce. Our findings suggest that infection heterogeneity may contribute to distinct clinical trajectories, but due to the small sample size and limited statistical power, these results should be interpreted as hypothesis-generating. Larger studies are warranted to determine whether specific infection types confer a higher risk of impaired decongestion or renal complications in CRS1.

This study has several limitations that warrant consideration. First, it was conducted at a single tertiary center with a relatively limited sample size, which may restrict the generalizability of the findings and reduce the statistical power to detect modest differences, particularly in secondary outcomes. The limited number of infected patients may have reduced statistical power, increasing the possibility of a type II error and potentially obscuring clinically relevant associations. Second, its observational design cannot exclude residual confounding despite multivariable adjustment, especially regarding infection severity, treatment heterogeneity, and comorbidity burden. Third, Incomplete follow-up and missing data reduced the effective sample size of the adjusted analyses, particularly for the MAKE-30 model, which may have introduced selection bias. Because missingness primarily reflected incomplete 30-day outcome ascertainment rather than isolated missing baseline covariates, multiple imputation was not considered appropriate, and complete-case analysis was performed. Fourth, infection was defined by clinical suspicion and antibiotic initiation, without systematic microbiological confirmation, and encompassed heterogeneous etiologies (pulmonary, urinary, soft tissue, and others) that may exert different prognostic effects. The lack of systematic microbiological confirmation may have resulted in misclassification of infection status, potentially attenuating true associations. Fifth, the analysis was restricted to short-term outcomes at 10 and 30 days, which may not reflect the longer-term impact of infection on decongestion trajectories, kidney recovery, or cardiovascular events in CRS1 patients. Sixth, the study lacks external validation in an independent cohort, and replication in larger, multicenter studies is needed to confirm that infection is not an independent determinant of decongestion or MAKE in this population. Additionally, several biomarkers, including CA-125, had substantial missing data, limiting statistical power and the reliability of between-group comparisons.

This study also has several important strengths. First, it focused on a well-defined cohort of patients with CRS1 and infection. Second, decongestion was assessed using predefined, clinically meaningful criteria, ensuring consistent evaluation across participants. Third, the use of multivariable models adjusted for major clinical confounders, complemented by sensitivity analyses, strengthens the robustness of the findings. Fourth, by incorporating kidney-centered outcomes such as MAKE and its components, the study provides a more comprehensive understanding of the kidney consequences of acute decompensated heart failure in the context of infection. Finally, the findings have direct clinical relevance, as they suggest that infection, despite being a common precipitant, does not independently compromise decongestion or short-term kidney outcomes, thereby supporting the safe and continued use of diuretics in this high-risk group under careful monitoring.

Clinically, these findings suggest that while infection may contribute to hemodynamic instability and greater biochemical derangements, the adjusted models underscore that short-term MAKE outcomes are not independently determined by infection but may instead be influenced by the cumulative burden of comorbidities and baseline renal reserve.

## Conclusions

Infection is a frequent trigger of CRS1 and is associated with greater baseline congestion, but it did not independently affect decongestion success or short-term kidney outcomes. Diuretic therapy remains effective in this setting under careful monitoring.

## Supporting information

S1 Fig Percentage of decongestion by origin of infection. (PDF)

## References

[1] Arrigo M, Jessup M, Mullens W, Reza N, Shah AM, Sliwa K, et al. Acute heart failure. Nat Rev Dis Primers. 2020;6(1):16. doi: 10.1038/s41572-020-0151-7 32139695 PMC7714436

[2] Ogbemudia EJ, Obasohan AO. Association between Common Etiologies and Precipitants of Acute Decompensated Heart Failure. Niger Med J. 2019;60(3):113–6. doi: 10.4103/nmj.NMJ_63_19 31543561 PMC6737795

[3] Bhatia MS, Sharda SC, Attri R, Pannu AK, Dahiya N. Correlation of mortality with Pro-BNP and precipitating factors of acute heart failure in patients presenting to a medical emergency of tertiary care hospital: an observational study from north India. Eur Rev Med Pharmacol Sci. 2022;26(18):6459–68. doi: 10.26355/eurrev_202209_29745 36196696

[4] Jobs A, Simon R, de Waha S, Rogacev K, Katalinic A, Babaev V, et al. Pneumonia and inflammation in acute decompensated heart failure: A registry-based analysis of 1939 patients. Eur Heart J Acute Cardiovasc Care. 2018;7(4):362–70. doi: 10.1177/2048872617700874 28357890

[5] Ueda T, Kawakami R, Horii M, Sugawara Y, Matsumoto T, Okada S, et al. Noncardiovascular death, especially infection, is a significant cause of death in elderly patients with acutely decompensated heart failure. J Card Fail. 2014;20(3):174–80. doi: 10.1016/j.cardfail.2013.12.007 24361802

[6] Chávez-Iñiguez JS, Sánchez-Villaseca SJ, García-Macías LA. Síndrome cardiorrenal: clasificación, fisiopatología, diagnóstico y tratamiento. Una revisión de las publicaciones médicas. Arch Cardiol Mex. 2022;92(2):253–63. doi: 10.24875/ACM.20000183 34261129 PMC9005172

[7] Biegus J, Cotter G, Metra M, Ponikowski P. Decongestion in acute heart failure: Is it time to change diuretic-centred paradigm?. Eur J Heart Fail. 2024;26(10):2094–106. doi: 10.1002/ejhf.3423 39169731

[8] de la Espriella R, Santas E, Zegri Reiriz I, Górriz JL, Cobo Marcos M, Núñez J. Quantification and treatment of congestion in heart failure: A clinical and pathophysiological overview. Nefrologia (Engl Ed). 2021;:S0211-6995(21)00114-4. doi: 10.1016/j.nefro.2021.04.006 36153911

[9] Chávez-Iñiguez JS, Ibarra-Estrada M, Sánchez-Villaseca S, Romero-González G, Font-Yañez JJ, De la Torre-Quiroga A, et al. The Effect in renal function and vascular decongestion in type 1 cardiorenal syndrome treated with two strategies of diuretics, a pilot randomized trial. BMC Nephrol. 2022;23(1):3. doi: 10.1186/s12882-021-02637-y 34979962 PMC8722345

[10] Zarbock A, Nadim MK, Pickkers P, Gomez H, Bell S, Joannidis M, et al. Sepsis-associated acute kidney injury: Consensus report of the 28th Acute Disease Quality Initiative workgroup. Nat Rev Nephrol. 2023;19(6):401–17. doi: 10.1038/s41581-023-00683-3 36823168

[11] Palazzuoli A, Ruocco G, Ronco C, McCullough PA. Loop diuretics in acute heart failure: Beyond the decongestive relief for the kidney. Crit Care. 2015;19(1):296. doi: 10.1186/s13054-015-1017-3 26335137 PMC4559070

[12] Ronco C, Haapio M, House AA, Anavekar N, Bellomo R. Cardiorenal syndrome. J Am Coll Cardiol. 2008;52(19):1527–39. doi: 10.1016/j.jacc.2008.07.051 19007588

[13] Martens P, Nijst P, Mullens W. Current approach to decongestive therapy in acute heart failure. Curr Heart Fail Rep. 2015;12(6):367–78. doi: 10.1007/s11897-015-0273-5 26486631

[14] Kellum JA, Lameire N. Diagnosis, evaluation, and management of acute kidney injury: A KDIGO summary (Part 1). Crit Care. 2013;17(1):204. doi: 10.1186/cc11454 23394211 PMC4057151

[15] Levey AS, Stevens LA, Schmid CH, Zhang YL, Castro AF, Feldman HI, et al. A new equation to estimate glomerular filtration rate. Ann Intern Med. 2009;150(9):604–12. doi: 10.7326/0003-4819-150-9-200905050-00006 19414839 PMC2763564

[16] Billings FT, Shaw AD. Clinical trial endpoints in acute kidney injury. Nephron Clin Pract. 2014;127(1–4):89–93. doi: 10.1159/000363725 25343828 PMC4480222

[17] Zarbock A, Forni LG, Koyner JL, Bell S, Reis T, Meersch M, et al. Recommendations for clinical trial design in acute kidney injury from the 31st acute disease quality initiative consensus conference. A consensus statement. Intensive Care Med. 2024;50(9):1426–37. doi: 10.1007/s00134-024-07560-y 39115567 PMC11377501

[18] Negi S, Koreeda D, Kobayashi S, Iwashita Y, Shigematu T. Renal replacement therapy for acute kidney injury. Ren Replace Ther. 2016;2(1). doi: 10.1186/s41100-016-0043-1

[19] von Elm E, Altman DG, Egger M, Pocock SJ, Gøtzsche PC, Vandenbroucke JP, et al. The Strengthening the Reporting of Observational Studies in Epidemiology (STROBE) statement: Guidelines for reporting observational studies. J Clin Epidemiol. 2008;61(4):344–9. doi: 10.1016/j.jclinepi.2007.11.008 18313558

[20] Benchimol EI, Smeeth L, Guttmann A, Harron K, Moher D, Petersen I, et al. The REporting of studies Conducted using Observational Routinely-collected health Data (RECORD) statement. PLoS Med. 2015;12(10):e1001885. doi: 10.1371/journal.pmed.1001885 26440803 PMC4595218

[21] Alvarez PA, Briasoulis A, Malik AH. Frequency and Impact of Infectious Disease Conditions Diagnosed During Decompensated Heart Failure Hospitalizations in the United States. Am J Cardiol. 2023;191:1–7. doi: 10.1016/j.amjcard.2022.12.001 36621054

[22] Alon D, Stein GY, Korenfeld R, Fuchs S. Predictors and outcomes of infection-related hospital admissions of heart failure patients. PLoS One. 2013;8(8):e72476. doi: 10.1371/journal.pone.0072476 24009684 PMC3751916

[23] Lee CH, Lin HW, Lin SH, Li YH. Impact of infection-related admission in patients with heart failure: A 10 years national cohort study. Sci Rep. 2023;13(1):6941. doi: 10.1038/s41598-023-34028-8 37117486 PMC10147930

[24] Bezati S, Velliou M, Ventoulis I, Simitsis P, Parissis J, Polyzogopoulou E. Infection as an under-recognized precipitant of acute heart failure: Prognostic and therapeutic implications. Heart Fail Rev. 2023;28(4):893–904. doi: 10.1007/s10741-023-10303-8 36897491 PMC9999079

[25] Schuermans A, Verbrugge FH. Decongestion (instead of ultrafiltration?). Curr Opin Cardiol. 2024;39(3):188–95. doi: 10.1097/HCO.0000000000001124 38362936

[26] Hollenberg SM, Warner SL, Ahmad T, Amin VJ, Bozkurt B, Butler J, et al. 2019 ACC Expert Consensus Decision Pathway on Risk Assessment, Management, and Clinical Trajectory of Patients Hospitalized With Heart Failure: A Report of the American College of Cardiology Solution Set Oversight Committee. J Am Coll Cardiol. 2019 Oct 15;74(15):1966–011. doi: 10.1016/j.jacc.2019.08.001 31526538

[27] Bilgeri V, Spitaler P, Puelacher C, Messner M, Adukauskaite A, Barbieri F, et al. Decongestion in acute heart failure - time to rethink and standardize current clinical practice?. J Clin Med. 2024;13(2):311. doi: 10.3390/jcm13020311 38256444 PMC10816514

[28] Cotter G, Davison B, Chioncel O. Enhanced decongestive therapy in patients with acute heart failure: JACC review topic of the week. J Am Coll Cardiol. 2024 Apr 2;83(13):1243–52. doi: 10.1016/j.jacc.2024.01.029 38538204

[29] Kapelios CJ, Vazir A, Lund LH, Filippatos G, Fang JC. Pharmacological options to relieve congestion in acute heart failure. Heart Fail Rev. 2025. doi: 10.1007/s10741-025-10548-5 40884709 PMC12618349

[30] Mentz RJ, Kjeldsen K, Rossi GP, Voors AA, Cleland JGF, Anker SD, et al. Decongestion in acute heart failure. Eur J Heart Fail. 2014;16(5):471–82. doi: 10.1002/ejhf.74 24599738 PMC4659502

[31] Villegas-Gutiérrez LY, Núñez J, Kashani K, Chávez-Iñiguez JS. Kidney replacement therapies and ultrafiltration in cardiorenal syndrome. Cardiorenal Med. 2024;14(1):320–33. doi: 10.1159/000539547 38810607

[32] Dilken O, Ince C, Kapucu A, Heeman PM, Ergin B. Furosemide exacerbated the impairment of renal function, oxygenation and medullary damage in a rat model of renal ischemia/reperfusion induced AKI. Intensive Care Med Exp. 2023;11(1):25. doi: 10.1186/s40635-023-00509-3 37121963 PMC10149155

[33] Guan C, Li C, Xu L, Che L, Wang Y, Yang C, et al. Hospitalized patients received furosemide undergoing acute kidney injury: the risk and prediction tool. Eur J Med Res. 2023;28(1):312. doi: 10.1186/s40001-023-01306-0 37660080 PMC10474726

[34] McCoy IE, Montez-Rath ME, Chertow GM, Chang TI. Estimated effects of early diuretic use in critical illness. Crit Care Explor. 2019;1(7):e0021. doi: 10.1097/CCE.0000000000000021 31440746 PMC6705600

[35] Chávez-Íñiguez JS, Ivey-Miranda JB, De la Vega-Mendez FM, Borges-Vela JA. How to interpret serum creatinine increases during decongestion. Front Cardiovasc Med. 2023;9:1098553. doi: 10.3389/fcvm.2022.1098553 36684603 PMC9846337

[36] Shen Y, Zhang W, Shen Y. Early diuretic use and mortality in critically ill patients with vasopressor support: A propensity score-matching analysis. Crit Care. 2019;23(1). doi: 10.1186/s13054-019-2309-9 PMC6329160 30630521

