# Supplementary material for: Impact of infection on decongestion and kidney outcomes in patients with cardiorenal syndrome type 1
Source: PLoS One. 2026 Aug 7;21(8):e0355608. doi: 10.1371/journal.pone.0355608 (PMC13450727; doi:10.1371/journal.pone.0355608)

Supplemental Figure. Percentage of decongestion by origin of infection.

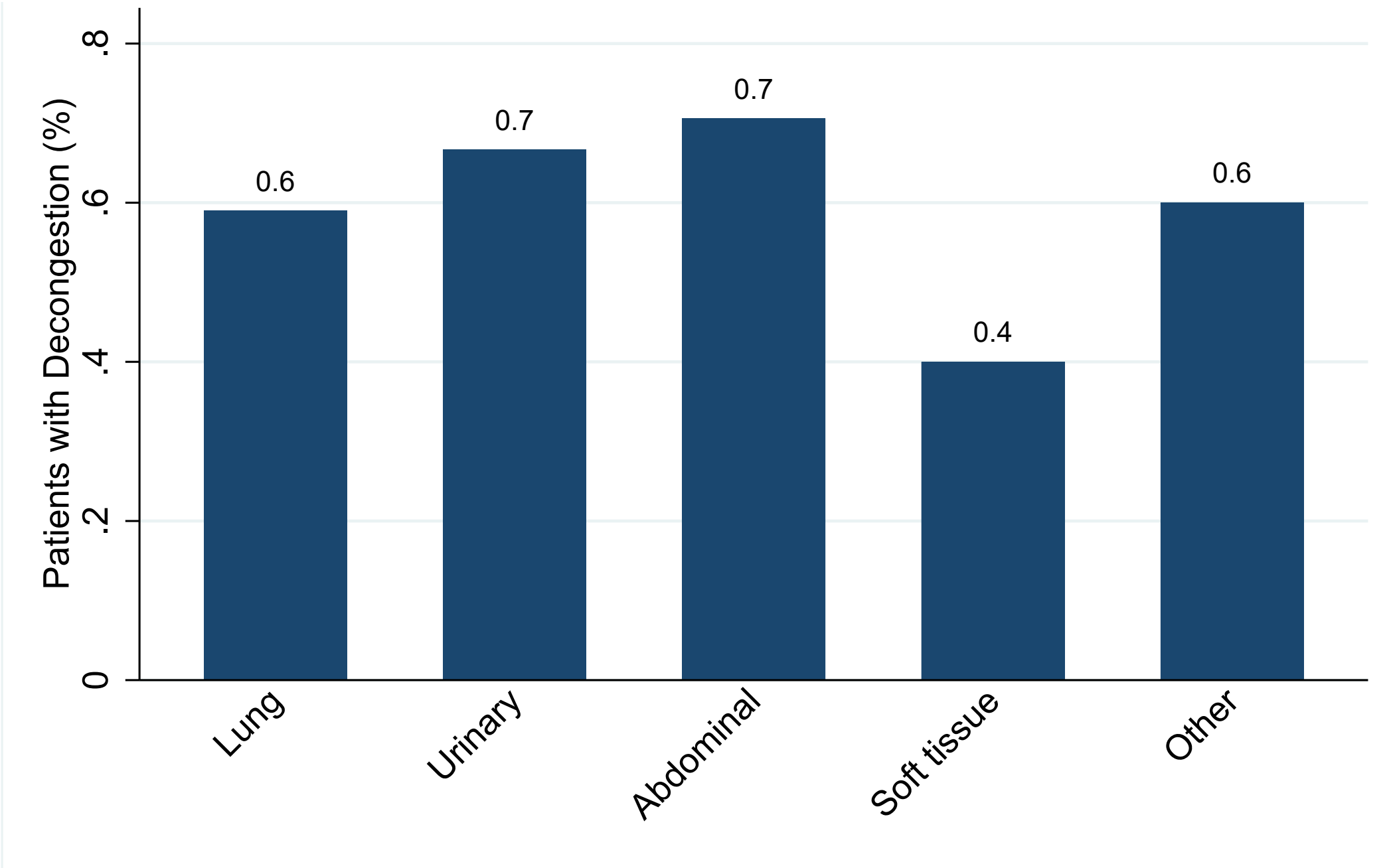

Supplement: Supplementary file 1 [file pone.0355608.s001.pdf]
